# Exploration of Digital Creativity: Construction of the Multiteam Digital Creativity Influencing Factor Model in the Action Phase

**DOI:** 10.3389/fpsyg.2022.822649

**Published:** 2022-05-16

**Authors:** Jing Zhang, Weilong Chen, Yuchun Xiao, Baohua Wang

**Affiliations:** ^1^School of Business Administration, Zhejiang Gongshang University, Hangzhou, China; ^2^Research and Innovation Center, Communication University of Zhejiang, Hangzhou, China; ^3^School of Economics, Zhejiang Gongshang University, Hangzhou, China

**Keywords:** cross-validation studies, influencing factor model, MTS theory, multiteam digital creativity, multiteam digital sharing

## Abstract

Based on multiteam system (MTS) theory and creativity theory, this study explores the influencing factor model of multiteam digital creativity (MTDC) in the action phase through two cross-validation studies, filling a theoretical gap and responding to the research call. Study 1 is a qualitative analysis method to fully explore the relevant influencing factors and enhance the theoretical saturation. Study 2 is an optimized DEMATEL method, known as the CL-WG DEMATEL analysis method, which cross-validates the new theoretical model and measures the centrality of the influencing factors. This study finds that the influence factor model of MTDC has eight major factors and distributes in four different levels. Further analysis shows that the three influences (team digital ability, multiteam digital sharing, and organizational digital resource matching) with the highest centrality of impact on MTDC all belong to the collective level, which indicates the uniqueness of the action phase of the performance episodes. The two cross-validation studies enhance the scientific validity of the new theoretical exploration. In addition, Theoretical and practical implications of the results are presented, and future directions for research are discussed.

## Introduction

With the rapid development of digital technologies such as big data, artificial intelligence, cloud computing, and the blockchain, the world has entered the digital era of rapid change and innovation (Yoo et al., 2010; Grover et al., 2020). Organizations and individuals are undergoing significant transformation as a result of digitization (Van Rensburg et al., 2021). Therefore, it is very important to adapt to digital trends and enhance digital ability (Shao et al., 2021). Creativity is the ability to flexibly adapt to change and solve complex situations, which is one of the most needed competencies in the digital era (Van Rensburg et al., 2021). At the intersection of creativity and digital technology is the emerging field of digital creativity (Lee and Chen, 2015). Thus, it is necessary to assess and study digital creativity to improve the organizations’ competencies (Yoo et al., 2012; Hoffmann et al., 2016; Van Rensburg et al., 2021). Digital creativity is defined as creativity that is expressed in various forms based on digital environments or driven by digital technologies (Lee et al., 2013; Lee and Chen, 2015). The literature on creativity is rich, but digital creativity in the organizational domain has yet to be more explored (Van Rensburg et al., 2021).

To accommodate this dynamic and complex environment, multiteam working models are increasingly prevalent (Turner et al., 2019; Mell et al., 2020). Meanwhile, digitization is also an important driver of the prevalence of multiteam working models (Kirkman and Mathieu, 2005; Maynard et al., 2012). The theory of multiteam systems (MTSs) provides a theoretical reference for organizations to carry out multiteam collaboration models, and academics are increasingly focusing on this topic (Luciano et al., 2018). MTS is defined as an interdependent system of multiple teams interacting to achieve a set of subgoals guided by a common goal (Mathieu et al., 2001).

Based on MTS theory and digital creativity theory, this paper argues that multiteam digital creativity (MTDC) is the formation of novel and practical ideas, products, processes, and services that are based on digital environments or driven by digital technologies and is a hybrid of multilevel synergistic evolution of intra-team or inter-team creativity. Furthermore, according to the MTS theory, the multiteam collaboration process includes the transition phase and action phase (Marks et al., 2001), referred to as performance episodes (Mathieu, 2006). Since different phases of performance episodes have unique characteristics, it is necessary to perform a targeted study. This study focuses on the action phase of MTS, which response to the theoretical call for studies on the specific phase in MTS contexts (Mathieu, 2006), filling a gap in the relevant research, further enriching MTS theory and creativity theory.

Previous studies have focused on creativity at the individual and team levels, but little is known about MTDC. A model study on the influencing factors of MTDC is a new field of creativity research, so it’s necessary to further improve theoretical saturation through multidimensional exploratory research. Based on the principle of theory-data matching, this study adopts two research methods to explore this new field. Study 1 is based on qualitative research methods such as rooting theory (Hoda et al., 2012), collecting valid concepts through semistructured in-depth interviews, further enhancing theoretical saturation based on continuous induction and revision, and initially constructing theoretical models that respond to phenomena and essences (Mello and Flint, 2009; Urquhart et al., 2010). Study 2 used the conceptual lattice-weighted group DEMATEL (CL-WG DEMATEL) analysis method to further cross-validate the new theoretical model and to analyze the importance of the influencing factors. The CL-WG DEMATEL analysis method is a more scientific and reasonable quantitative measure that takes into account multiple dimensions such as expert group opinion, expert weighting ratio, and the degree of interaction between influencing factors (Shi et al., 2016). Both study methods are based on a cognitive perspective, using an integrated decision-making approach and cross-validation to explore in-depth the framework of influencing factors in new areas, effectively enhancing the scientific and rigorous degree of exploratory research.

## Theoretical Background

### Multiteam Systems Theory

After MTS theory was proposed, more scholars have focused on this area and developed a rich theoretical study (Lei et al., 2022). Studies have shown that the effectiveness of MTS depends on factors such as cognition and motivation (Lanaj et al., 2013), leadership (DeChurch and Marks, 2006), behavioral processes (Van den Berg et al., 2014), multiteam collaboration (Davison et al., 2012), cross-border identity (Cuijpers et al., 2016), decision making (Waring et al., 2020), and risk appetite (Lanaj et al., 2018). For example, after reviewing the literature, Zaccaro et al. (2020) found that important influencing factors of multiteam effectiveness include leadership structure, cognition, internal and external coordination processes, emotional and motivational emergent states, MTS boundary states (internal or external), and team variability (geographical, functional, cultural, and normative). Some scholars also find that the social identity of multiteam systems will have negative effects in task complexity situations due to more individual depletion (Porck et al., 2019).

The effectiveness of MTS may also depend on the different phases of the performance episodes of the MTS (Mathieu, 2006). For example, the performance of MTS is also related to a more granular classification of the action phase (Torres et al., 2021). Therefore, the study of MTS requires targeted research for different stages. In addition, multiteam systems also include two key structural characteristics, namely, variability (diversity) and dynamism, and three key factors, including attribution needs, cognitive abilities, and affective states (Luciano et al., 2018). These key structural features and key factors will all likely affect the effectiveness of the multiteam system.

Overall, multiteam systems’ effectiveness depends on complex influencing factors, and the influencing factors include multiple dimensions, such as cognitive abilities at the individual level, team diversity at the team level, MTS boundary state at the multiteam level, and culture and norms at the organizational level. Scholars have called for more research on organizational behavior from the multiteam perspective (Mathieu et al., 2008; de Vries et al., 2022). And studies of MTS theory provide a good theoretical foundation for this study.

### Digital Creativity and Multiteam Digital Creativity

Digital creativity is a special form of creativity (Shao et al., 2021). Creativity refers to the formation of ideas, products, processes, and services that are novel and useful to an individual or team (Amabile et al., 1996). Creativity is the result of individual and contextual interactions (Woodman et al., 1993). With the rapid development of the digital economy, there is a growing interest in digital innovation and creativity. The journal *Digital Creativity*, published by the British scholar Owen Kwlly at the end of the 20th century, initiated the focus on digital creativity in some fields. One study found that despite the rapid growth of digitalization, organizations lack strategies to creatively use digital technology, which is called the “digital impasse.” The main reason for this dilemma is the lack of digital creativity (Shao et al., 2021). Digital creativity is becoming a very important competency (Van Rensburg et al., 2021).

Digital creativity is the creation of working in a digital environment and presenting computer and network processing skills in products. Digital creativity is closely related to digital tendencies, digital environment, professional skills, and other influencing factors (Lee et al., 2013). Digital creativity is a new way of exploring and presenting creativity using digital tools and technologies (Van Rensburg et al., 2021). Lee and Chen (2015) defined digital creativity as the diverse creativity that emerges from an individual, team, or organization driven by digital technologies. Digital creativity is defined as a new and useful idea or plan generated by employees to achieve better performance through the use of digital technology, which is a contextualized form of creativity and usually involve changes in products, services, and processes in organizational settings (Shao et al., 2021).

In addition, numerous scholars have studied creativity influencing factors. The three-factor model considers the influencing factors like expertise, creative skills, and intrinsic motivation (Amabile, 1988), and Woodman proposed an interaction model that suggests the interaction of individual-, team-, and organizational-levels factors affect creativity (Woodman et al., 1993). Regarding digital creativity, some scholars have found that individual-level influencing factors include professional skills, digital tendency (Lee et al., 2013), ambidextrous learning capabilities and digital knowledge (Shao et al., 2021), digital entrepreneurial opportunity, and IT (information technology) capabilities (Tang et al., 2022), team-level factors have team cognitive states, and digital project-specific cognitions (Hadjielias et al., 2021). Organizational level predictors comprise the digital environment (Lee et al., 2013), task variety (Shao et al., 2021), organizational readiness, digital capabilities and digital organizational culture (Zhen et al., 2021), orchestration, and coordinate mechanisms (Urbinati et al., 2021). From an ecosystems perspective, dynamic capabilities are critical to creativity (Abbate et al., 2022). In addition, transformational IT leadership and IT governance, and digital entrepreneurial opportunity are also predictors of digital creativity (Pittenger et al., 2022).

Overall, creativity research is rich, but digital creativity studies are relatively scarce and have only been focused on in the last decade. Moreover, digital creativity has not yet been studied from a multiteam systems theory perspective. Multiteam models are very common in the current digital era, and therefore scholars call for organizational issues to be studied from an MTS theory perspective (Mathieu et al., 2008; de Vries et al., 2022). MTDC is a new area of creativity study. This paper systematically explores the model of factors influencing MTDC from the perspective of MTS theory and digital creativity theory, filling a gap in the current research field, and as an urgent need to match the current boom of the digital economy and digital society.

## Study 1: Exploration of the Factors Influencing

### Method

This study used qualitative analysis methods such as rooted theory to fully explore new factors of theory and enhance theoretical saturation (Glaser, 1978; Hoda et al., 2012). In this study, methods such as online video or face-to-face semistructured interviews were used. The study was reviewed by the Zhejiang Gongshang University’s Research Committee and declared to comply with the university’s ethical and legal principles and guidelines.

### Participants and Procedure

To effectively improve the representativeness of the sample, the criteria of selecting interview subjects mainly followed the matching principle. Drawing on qualitative research methods such as rooting theory, three steps were adopted in this study. First, 15 middle and senior managers from 4 companies were selected as the preliminary interviewees, and 51 other managers were initially interviewed based on their recommendations. These managers are mostly managers of high-tech enterprises in which digital technology has been deeply embedded, so these managers have a more in-depth understanding of digital creativity.

Second, the relevant concepts were fully communicated with the interviewees according to the definition of the action stage in the performance episodes theory, so the interviewees could gain a deeper understanding of the study semantics of MTS and identify operational definitions and fitness criteria. According to multiteam systems theory, interviewees should manage two or more different teams with one or more common goals, and there is a strong interdependence between the teams in at least one aspect, in line with multiteam characteristics (Mathieu et al., 2001). At the same time, the selection should meet the core control criteria for the action phase: at least 2 teams collaborating on a common goal task by a specified deadline and plan, and at least 1 systematic monitoring, goal moderation, or support of responsive behavior.

Finally, 35 persons in charge or managers who met the operational criteria and adaptation principles were selected according to the preliminary interview results and interviewed in-depth for at least 1 h. It was clearly stated before the interview that the results would be used for academic research only. Information was confidential and names would be substituted in code form.

### Analysis

The coding in this paper includes the processes of excerpting, coding, and categorizing and forms a three-level coding based on the analysis and categorization. This method mainly adopts the interview coding procedure introduced by Glaser (1978).

#### First-Level Coding

The excerpting, coding, and categorizing were completed by three study team members. To improve the reliability and validity, those concepts that reached a consensus were put into the initial concept base, while those concepts that did not reach a consensus concept were decided collectively. Interviews and coding were performed in parallel individually. Then, based on the next batch of interviews, invalid concepts were eliminated and clustered to form valid concepts. Statistics were conducted based on the coding, and valid concepts with higher mention frequency were screened out and finally summarized into valid codes. Some valid codes in action are shown in Table 1.

**TABLE 1:** Coding database of some interview contents in the action phase.

Effective concepts	Frequency	Overview of some of the interviews
IT capabilities	18	Master basic IT knowledge and IT skills, and have a good ability to explore IT applications.
Individual characteristics	21	Personal tendency to explore new things, like new winds, and love digital innovation.
Task adaptation	12	Tasks match individual characteristics and workload. Too much task pressure, information load, or too tight time is not beneficial for innovation.
Network resources	23	Rich social resources and access to novel and useful information from multiple sources, especially digital innovation resources.
Cross-border capabilities	12	Leaders are able to coordinate well across multiple teams, especially between digital technology teams and traditional teams.
Management capabilities	18	Ability to choose the right leadership style to manage and motivate everyone.
Identification ability	15	Accurately and quickly identifies innovation-related information in the internal and external digital environment.
Absorptive capacity	19	Ability to quickly identify, absorb, and use external knowledge.
Ambidextrous competence	25	Restructuring, integrating, or transforming existing resources and new digital resources; successfully implementing new and complex resource combinations.
Task conflict	19	When setting goals, assigning tasks, or analyzing problems, the group sometimes heatedly discusses and sometimes argues.
Relationship conflict	9	There are conflicts in emotional relationships and do not see eye to eye with each other.
Process conflict	10	Disagreement on the way to work and the process.
Interaction mechanism	19	Interaction between multiple teams is very efficient through digital technology.
Monitoring mechanism	15	The evaluation of goals and tasks is clear and standardized while retaining some flexibility.
Feedback mechanism	12	Ability to give quick and timely feedback, and collaborate effectively.
Information sharing	20	Often things are understood differently and there are differences in information between teams. Information sharing mechanism is important.
Knowledge sharing	27	There are differences in knowledge, such as explicit knowledge and implicit knowledge. Knowledge sharing benefits innovation.
Resource sharing	17	There are differences in resources between multiple teams, especially digital resources, and it is important to support each other.
Risk balancing	16	With effective control between opportunities and risks, the organization does not abandon new goals for the sake of some risks.
Process monitoring	11	Monitoring, support, and feedback through organizational management and digital technology.
Controlled results	7	Develop along with the organization’s strategic goals. Allowing for some exploratory mistakes, the organization’s strategy supports digital development.
Digital infrastructure resources	17	Digital infrastructure is strong support for organizational digital creativity.
Digital human capital	20	The organization can provide strong support and facilitation when more people with various skills are needed, especially digital talent.
Platform and ecological resources	18	It is important to have a digital platform and ecosystem that is compatible with the organization; if you do not proactively integrate you will miss the windfall.

#### Second-Level Coding

The main categories of the action phase of MTDC were clustered into eight categories through interview summarization, theory reference, industry and theory expert opinions, and clustering and categorization based on multiple sources of reference. These categories include individual-task matching, multiteam leadership; team digital ability, team conflict; multiteam collaboration mode, multiteam digital sharing; organizational fitness, digital resource matching. Some of the secondary codes are shown in Table 2.

**TABLE 2:** Main category library of the second-level coding in the action phase.

Main categories	Effective concepts	Main category connotations
Individual-task matching	IT capabilities Individual characteristics Task adaptation	The members have the personality, digital professional skills, and IT capabilities that are a good match for the tasks they undertake.
Multiteam leadership	Network resources Cross-border capabilities Management capabilities	Leadership’s management style, resources, and capabilities promote creativity.
Team digital ability	Identification ability Absorptive capacity Ambidextrous competence	Reconstruct, integrate and transform existing and new digital resources to achieve new complex resource combinations. The dynamic ability to absorb and identify knowledge, information, and the environment is critical to digital innovation (Zahra et al., 2006).
Team conflict	Task conflict Relationship conflict Process conflict	A perceptual process arising from differences or dissonance in goals, perceptions, and visions among team members, classified as task conflict (TC), relationship conflict (RC), and process conflict (PC) (Jehn and Mannix, 2001).
Multiteam collaboration mode	Interaction mechanism Monitoring mechanism Feedback mechanism	Normative, shared patterns of behavior among multiple teams, including implicit and explicit, horizontal and vertical, and other collaborations (Davison et al., 2012).
Multiteam digital sharing	Information sharing Knowledge sharing Resource sharing	The degree of shared assistance between multiple teams in digital information, knowledge, and digital resources. The ease and effectiveness of supporting mutual assistance in a digital open environment.
Organizational fitness	Risk balancin Process monitoring Controlled results	Adaptability and resilience of the organization to goals, processes, and outcomes. The organization can respond to rapidly changing and complex environments.
Digital resource matching	Digital infrastructure resources Digital human capital Platform and ecological resources	The digital infrastructure, digital platforms, and ecosystems that are available in the organization are the foundation. The combination with human resources is the guarantee of digital creativity.
		

#### Third-Level Coding

Based on creativity theory and MTS theory, a theoretical structure model was established by logically analyzing the eight second-level main categories. The four key categories of MTDC in the action phase were mined according to the hierarchical structure, which are the individual, team, multiteam, and organizational levels.

### Results

**FIGURE 1:**
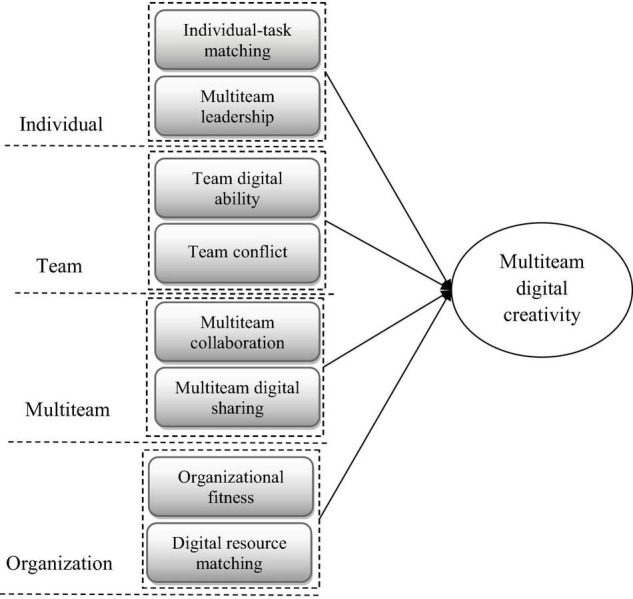
Model of the factors influencing MTDC in the action phase.

Referring to Mathieu and Marks’ performance episodes theory, the action phase focuses on continuous monitoring of multiteam goals and systems, mutual support among teams, mutual assistance and synergy in task achievement, and communicate feedback to ensure the successful completion of multiteam and organizational goals (Mathieu et al., 2001). With reference to the performance episodes theory and the coding results stated above, this study found a model of MTDC influencing factors in the action phase that includes four levels, specifically include the individual level (individual-task matching, multiteam leadership), team level (team digital ability, team conflict), multiteam level (multiteam collaboration mode, multiteam digital sharing), and organizational level (organizational fitness, digital resource matching). The model is shown in Figure 1.

Combining Tables 1, 2 and by integrating the analysis, the four most frequently aggregated expressions are multiteam leadership, team digital ability, multiteam digital sharing, and digital resource matching. These four main categories are distributed at exactly four different levels. This is an interesting result. Moreover, among these four second-level main categories, the most talked-about first-level effective concepts are network resources, ambidextrous, knowledge sharing, and digital human capital, respectively. These results provide some theoretical references for organizational practice.

## Study 2: Cross-Validation and Importance Analysis

### Method

Study 2 adopted the conceptual lattice-weighted group DEMATEL analysis method (Acronym CL-WG DEMATEL method). The DEMATEL analysis method is a method for identifying and evaluating complex relationships between influencing factors (Shi et al., 2016). Now it has been widely cited by scholars. However, the traditional DEMATEL method has some limitations, such as the one-sidedness of a single expert opinion. To solve this problem, some scholars have proposed the combination of DEMATEL and group decision making, in which multiple experts make collective decisions, thus enhancing the accuracy of the relational clustering process. Although the approach combined with group decision solves the single-expert one-sided problem, there are still problems, such as many differences in learning, experience, values, and education among experts, which may affect the final clustering process and judging results. Therefore, a better complementary method for assigning weights to expert scores is the concept lattice technique (Wille, 1983, 1992). The process of constructing a concept lattice is a process of concept clustering, and the concept lattice method allows clustering to obtain expert weight coefficients. The concept lattice technique effectively solves the drawback that the traditional DEMATEL research method cannot integrate the degree of expert influence. Based on Study 1, this study applies the optimized means—CL-WG DEMATEL method—which is a more rigorous and scientific method to analyze the importance of the influencing factors of MTDC in a comprehensive way (Shi et al., 2016).

### Participants and Procedure

The calculation steps of the optimized method are briefly described as follows.

First, determine the set of impact factors for the outcome variable: *X* = {*X*_1_, *X*_2,_ …, *X*_*n*_}.

Second, the direct relationship between the influencing factors is obtained after the integrated evaluation. University experts or industry mentors who are familiar with organizational behavior are invited to evaluate. The direct influence matrix is:


$$z=\left[\begin{array}{cccc} 0 & Z_{12} & \ldots & Z_{1J}\\ Z_{21} & 0 & \ldots & Z_{2J}\\ \ldots & \ldots & 0 & \ldots \\ Z_{i1} & Z_{i2} & \ldots & 0 \end{array}\right]$$

Third, calculate the expert weight coefficients and give the new direct influence matrix. First, cluster the results of the evaluation of a class of factors according to *r* experts with the same weight of similar experts. Then, obtain the influence coefficient among the factors according to the expert weights: $z_{ij}^{\prime }=\sum_{i=1}^{p}g_{i}Z_{ij}\left(i,j=1,2,\ldots ,n\right)$. Finally, the direct impact matrix after optimization of the weighting factors is given as:


$$z=\left[\begin{array}{cccc} 0 & Z_{12}^{\prime } & \ldots & Z_{1J}^{\prime }\\ Z_{21}^{\prime } & 0 & \ldots & Z_{2J}^{\prime }\\ \ldots & \ldots & 0 & \ldots \\ Z_{i1}^{\prime } & Z_{i2}^{\prime } & \ldots & 0 \end{array}\right]$$

Fourth, calculate the comprehensive impact matrix *W*.

If: $g=1/\max\left(\sum_{j=1}^{\text{n}}Z_{ij}^{\prime }\right)$, where *N=gZ*,

Then: *W* $=\lim_{k\to \infty }\left(N+N^{2}+\ldots +N^{K}\right)=N{\left(1-N\right)}^{-1}$

Finally, the degree of influence, degree affected, degree of centrality, and degree of causation are calculated according to the comprehensive influence matrix.

DI (the degree of influence): $f_{i}=\sum_{i=1}^{\text{n}}t_{ij}\left(i=1,2,\ldots ,n\right)$

DA (degree affected): $e_{i}=\sum_{j=1}^{\text{n}}t_{ji}\left(i=1,2,\ldots ,n\right)$

DCT (degree of centrality): *r*_*i*_ = *f*_*i*_ + *e*_*i*_

DCS (degree of causation): *z*_*i*_ = *f*_*i*_−*e*_*i*_

### Analysis

In this study, the influencing factors of MTDC are derived from a previous qualitative analysis. Here, the calculation process based on the CL-WG DEMATEL method is as follows:

First, determine the set of impact factors *X* = {*X*_1_, *X*_2,_ …, *X*_*n*_}

Second, obtain the direct relationships between the influencing factors. Six university experts or industry mentors judge the direct relationships between the factors. The evaluations are divided into five grades from 0 (none) to 5 (strong). The partially initialized direct influence matrix is:


$$Z_{1}=\left[\begin{array}{c} 0\;4\;4\;4\;3\;4\;2\;4\\ 4\;0\;4\;3\;4\;5\;4\;4\\ 4\;4\;0\;4\;5\;5\;4\;4\\ 3\;4\;4\;0\;3\;4\;3\;4\\ 3\;4\;5\;4\;0\;5\;3\;4\\ 4\;4\;5\;4\;5\;4\;4\;5\\ 3\;4\;4\;4\;4\;3\;0\;3\\ 5\;4\;5\;3\;4\;4\;3\;0 \end{array}\right]$$

$$Z_{2}=\left[\begin{array}{c} 0\;4\;4\;4\;3\;4\;3\;4\\ 5\;0\;4\;5\;4\;5\;2\;4\\ 4\;4\;0\;4\;4\;4\;3\;5\\ 3\;4\;3\;0\;3\;4\;4\;4\\ 3\;4\;4\;3\;0\;4\;3\;4\\ 5\;4\;4\;4\;4\;0\;3\;4\\ 4\;4\;5\;4\;4\;3\;0\;3\\ 4\;4\;4\;5\;4\;3\;4\;0 \end{array}\right]$$

$$Z_{3}=\left[\begin{array}{c} 0\;2\;4\;4\;4\;5\;3\;4\\ 5\;0\;5\;3\;4\;5\;4\;3\\ 4\;4\;0\;4\;4\;5\;3\;4\\ 2\;2\;2\;0\;3\;2\;4\;5\\ 3\;4\;4\;4\;0\;4\;1\;3\\ 4\;5\;5\;4\;5\;0\;3\;4\\ 1\;2\;3\;3\;2\;2\;0\;2\\ 4\;4\;4\;5\;4\;4\;3\;0 \end{array}\right]$$

Third, calculate the expert weighting coefficients and determine the new direct relationship matrix:


$$Z=\left[\begin{array}{cccccccc} 0 & 3.21 & 4 & 4 & 3.2 & 3.79 & 5.4 & 4.04\\ 4.45 & 0 & 4 & 3.43 & 3.57 & 4.5 & 3.21 & 3.8\\ 4.2 & 4 & 0 & 4 & 4.04 & 3.61 & 2.6 & 4.04\\ 2.96 & 3.83 & 3.57 & 0 & 3.04 & 3.94 & 3.21 & 4.04\\ 3.2 & 4 & 4.04 & 3.5 & 0 & 4.2 & 2.57 & 3.8\\ 4.04 & 4.19 & 4.5 & 4 & 4.5 & 0 & 3.19 & 4.19\\ 2.5 & 3.36 & 3.43 & 3.2 & 3.21 & 2.96 & 0 & 1.94\\ 4.2 & 4 & 4.21 & 4 & 4 & 4.21 & 3.2 & 0 \end{array}\right]$$

Fourth, calculate the integrated impact matrix *W*, where *g* = 1/28.62. The normalized direct relationship matrix is:


$$\begin{array}{c} N=[\begin{array}{cccc} 0 & 0.1123 & 0.1398 & 0.1398\\ 0.1573 & 0 & 0.1398 & 0.1198\\ 0.1468 & 0.1398 & 0 & 0.1398\\ 0.1035 & 0.134 & 0.1248 & 0\\ 0.1118 & 0.1398 & 0.1411 & 0.1223\\ 0.1411 & 0.1465 & 0.1573 & 0.1398\\ 0.0874 & 0.1173 & 0.1198 & 0.1118\\ 0.1468 & 0.1398 & 0.1473 & 0.1398 \end{array}\\ \begin{array}{cccc} 0.1118 & 0.1323 & 0.1887 & 0.1411\\ 0.1248 & 0.1573 & 0.1123 & 0.1328\\ 0.1411 & 0.1262 & 0.0909 & 0.1411\\ 0.1062 & 0.1378 & 0.1123 & 0.1411\\ 0 & 0.1468 & 0.0899 & 0.1328\\ 0.1573 & 0 & 0.1116 & 0.1465\\ 0.1123 & 0.1035 & 0 & 0.068\\ 0.1398 & 0.1473 & 0.1118 & 0 \end{array}] \end{array}$$

The transformed combined impact matrix is:


$$\begin{array}{c} W=[\begin{array}{cccc} 1.2242 & 1.3645 & 1.4337 & 1.3664\\ 1.3584 & 1.2587 & 1.4297 & 1.3467\\ 1.3296 & 1.3603 & 1.2848 & 1.3413\\ 1.2159 & 1.2740 & 1.3106 & 1.1376\\ 1.2572 & 1.3139 & 1.3600 & 1.2813\\ 1.4067 & 1.4499 & 1.5084 & 1.4240\\ 1.0296 & 1.0833 & 1.1219 & 1.0631\\ 1.3802 & 1.4128 & 1.4677 & 1.3929 \end{array}\\ \begin{array}{cccc} 1.3216 & 1.4051 & 1.2842 & 1.3589\\ 1.3280 & 1.4211 & 1.2202 & 1.3510\\ 1.3197 & 1.3762 & 1.1836 & 1.3373\\ 1.2135 & 1.3009 & 1.1263 & 1.2557\\ 1.1515 & 1.3441 & 1.1393 & 1.2847\\ 1.4139 & 1.3498 & 1.2737 & 1.4235\\ 1.0462 & 1.0922 & 0.8676 & 1.0234\\ 1.3696 & 1.4455 & 1.2465 & 1.2644 \end{array}] \end{array}$$

### Results

According to the above calculation method and process, the results of DI, DA, DCT, and DCS in the action phase are shown in Table 3, where the orders are based on the DCT numbering.

**TABLE 3:** Calculation results of each degree in the action phase.

Influence factor	DI	DA	DCT	DCS	Order
Individual-task matching	10.7587	10.2018	20.9605	0.5569	5
Multiteam leadership	10.7137	10.5174	21.2312	0.1963	4
Team digital ability	10.5329	10.9169	21.4497	−0.384	2
Team conflict	9.8345	10.3533	20.1879	−0.5188	7
Multiteam collaboration mode	10.1321	10.1639	20.2961	−0.0318	6
Multiteam digital sharing	11.25	10.7349	21.9849	0.5151	1
Organizational fitness	8.3272	9.3415	17.6688	−1.0143	8
Digital resource matching	10.9795	10.2989	21.2784	0.6806	3

Table 4 shows the differences in the centrality of MTDC in the action phase based on the results of the interaction validation study.

**TABLE 4:** Difference in the centrality of MTDC in the action phase.

Influence factors		Action phase
Individual	Individual-task matching	✮✮
	Multiteam leadership	✮✮✮
Team	Team digital ability	✮✮✮✮
	Team conflict	✮
Multiteam	Multiteam collaboration mode	✮✮
	Multiteam digital sharing	✮✮✮✮
Organization	Organizational fitness	✮
	Digital resource matching	✮✮✮

*The number “✮” indicates the centrality ranking of the influencing factors. The more “✮” the higher the ranking.*

According to the degree of centrality, this study finds that the higher centrality of the MTDC influencing factors are multiteam leadership, team digital ability, multiteam digital sharing, and digital resource matching. Among them, multiteam digital sharing is the most central influencing factor and belongs to the multi-team level. The four core factors in the action phase are consistent with Study 1 and are distributed in four different levels.

## Discussion

This paper uses the cross-validation analysis method, Study 1 is the qualitative method, and Study 2 is the optimized CL-WG DEMATEL quantitative method. According to two studies, the model of the MTDC influence factor is multi-level, which is consistent with the research on the model of creativity influence factors (Gu et al., 2017). The main difference is that the multiteam level has become a key dimension, this is due to the increasing prevalence of current multiteam working patterns (de Vries et al., 2022). In new product innovation, the multiteam system has been increasingly adopted as an organizational form Lei et al. (2022).

Further comparative analysis reveals that the four influences with the highest centrality ranking are distributed at four different levels. This result implies that the influencing factors at the action to MTDC are diverse. Multiple levels of factors come together to contribute to MTDC. Previous research has shown that creativity is the result of multi-level interactions (Woodman et al., 1993). Digital creativity is influenced by multiple levels of interaction, including individual, team, and organizational (Gu et al., 2017). The difference is that the multiteam level factors play a more important role.

According to the results of the centrality calculation, team digital ability, multiteam digital sharing, and digital resource matching are particularly important for MTDR in the action phase. This may be attributed to the fact that multiteam systems are more mature in the action phase than in the transition phase, and they know each other. Therefore, the collective level at this phase is more important. Further analysis found that the top ranking in centrality is multiteam digital sharing, which belongs to the multiteam level. This result indicates that multiteam heterogeneity brings more creative thinking, but stimulating creativity in the action phase requires turning heterogeneity into collaborative support. Moreover, sharing digital information and knowledge can provide better openness, convenience, and adaptability for the multiteam system. These are beneficial to the focus of the action phase, i.e., the mutual synergy and communication feedback between teams (Mathieu et al., 2001). Therefore, the organization should pay special attention to multiteam digital sharing during the action phase.

In addition, the second important collective factor is team digital ability. Team digital ability includes the team ambidextrous capability, absorptive capacity, and recognition capacity. Due to the self-growing nature of digital technologies, the continuous dynamics of digital environments, and the complex combinations of Multi-competence are critical to reconfigure, integrate, and transform digital resources and achieve multiple digital innovation balances (O’Reilly and Tushman, 2013; Nylen and Holmstrom, 2019).

The third important collective factor is digital resource matching. Digital resource matching refers to the adaptability of digital resources to MTDC at the organizational level, which comprises digital infrastructure resources, digital human capital, and platform and ecological resources. Digital resource matching at the organizational level is the basis for digital product innovation, process innovation, organizational innovation, and business model innovation, as well as an important support for digital creativity. Digital infrastructure has an “enabling effect” on digital process innovation, enabling rapid formation, modification, and reconstruction of digital products and facilitating the implementation of digital innovation (Van Rensburg et al., 2021). The flexibility, openness, and availability of digital platforms and ecosystems, as well as the forward-looking orientation of technologies such as cloud computing and big data, have become central to the digital innovation activities of companies (De Reuver et al., 2018). Therefore, digital resources such as platform ecology are very critical (Abbate et al., 2022).

## Limitations

This study has a few limitations. First, the limitation of this study is that the results of the interviews are not conducted using an empirical research approach. This study followed the principle of matching theory and data and adopts qualitative analysis and CL-WG DEMATEL method. The cross-validation of both methods improves the reliability and validity of the study. In future studies, methods such as empirical methods can be used to increase the saturation of theory.

Second, the interviewees are mainly from some companies in coastal China, the sample is still rather one-sided. Although the selected companies are at the forefront of digital construction and can better reflect the typical situation of MTDR. Meanwhile, this study also used three steps to screen the sample to improve the representative. For future studies, a wider range of companies and a wider range of countries could be selected to improve the representativeness.

Finally, due to space limitations, this paper only studied the action phase and don’t conduct a complete study of performance episodes. In the future, a comparative study of the transition and action phases could be conducted. Overall, this paper enriches theoretical studies of MTS theory and digital creativity theory and responds to the organizational development needs of the digital era.

## Conclusion and Implication

Through the qualitative method of Study 1 and the CL-WG DEMATEL method of Study 2, this paper summarizes a multilevel framework of factors influencing MTDC in the action phase. Using the CL-WG DEMATEL method, this study finds that there are also differences in the impact effects of this diversity, which helps us to be able to better grasp the understanding of the model of the factors influencing MTDC in the action phase, and enriches a theoretical reference for high-tech enterprises to improve MTDC.

First, Study 1 through interviews, questionnaires, and coding finds that MTDC depends on influencing factors at the individual level, team level, multiteam level, and organizational level. In the action phase, individual-level factors include individual-task matching and multiteam leadership, team-level factors include digital ability and team conflict, multiteam level factors include multiteam collaboration mode and multiteam digital sharing, organizational level factors include organizational fitness and digital resource matching. Stimulating multi-team digital creativity is a complex system. Therefore, organizations should intervene at multiple levels to fully stimulate MTDC. For example, at the individual level, the organization should focus on developing the IT capabilities of employees to match the requirements of digital innovation. At the team level, the organization should strive to enhance ambidextrous competencies and reorganize, and thus successfully implement, new complex resource combinations (Shao et al., 2021). At the multiteam level, organizations should strive to improve knowledge sharing among teams, such as building a shared mental model among multi-teams (Lei et al., 2022). At the organizational level, it should build a platform ecosystem and other adapted digital resources. MTDC requires synergistic cooperation and multifrequency resonance of multiple influence levels.

Second, Study 2 adopted further interactive validation exploration, and the results showed that the four factors with higher centrality were multiteam leadership, team digital ability, multiteam digital sharing, and digital resource matching, respectively. These results are consistent with the coding summary results of Study 1. Further analysis reveals that the three factors with the highest centrality belong to the collective level, with multiteam digital sharing being the highest. This indicates that MTDC in the action phase is more dependent on collective-level influences, especially multiteam factors. This finding may be distinct from the transition phase of the performance episodes. Therefore, in the action phase, organizations need to focus more on digital resources, collaboration, and information sharing at the collective level rather than on individual-level capabilities, especially at the multi-team level.

Third, this study finds that the influencing factors of MTDC are both similar but significantly different compared to traditional organizational creativity. MTDC focuses more on digital characteristics, such as team digital ability, multiteam digital sharing, and digital resource matching. MTDC also focuses on MTS-based perspectives, such as multiteam leadership, multiteam collaboration model, and multiteam digital sharing. Therefore, to stimulate MTDC during the action phase, it is necessary to focus on both the original characteristics of MTS and the uniqueness of digital creativity. Because DTMC is the intersection of digital, creativity, and MTS (Van Rensburg et al., 2021; Lei et al., 2022). In the digital era, an increasing number of organizations adopt the multiteam model to carry out digital innovation work, and managers should fully understand the influencing factors of MTDC to adopt appropriate management strategies to further stimulate organizational creativity.

Finally, this study further enriches the approach to exploring new research areas by using a cross-validation research approach of Studies 1 and 2. Study 1 explores the impact factor of the new field, and Study 2 validates the results of Study 1 and calculates the importance of the impact factor. Thus the two approaches are mutually validating and complementary to each other, which can effectively improve the reliability and validity of exploratory studies. With both studies, this research further enriches the MTS theory and creativity theory literature.

## Data Availability Statement

The original contributions presented in the study are included in the article/supplementary material, further inquiries can be directed to the corresponding author.

## Ethics Statement

Ethical review and approval was not required for the study on human participants in accordance with the local legislation and institutional requirements. Written informed consent from the patients/participants was not required to participate in this study in accordance with the national legislation and the institutional requirements.

## Author Contributions

YX and WC: conceptualization, methodology, writing – review and editing, project administration, and funding acquisition. WC and JZ: software, formal analysis, investigation, data curation, writing – original draft preparation, and visualization. YX: supervision. WC, JZ, and BW: validation. All authors have read and agreed to the published version of the manuscript.

## Conflict of Interest

The authors declare that the research was conducted in the absence of any commercial or financial relationships that could be construed as a potential conflict of interest.

## Publisher’s Note

All claims expressed in this article are solely those of the authors and do not necessarily represent those of their affiliated organizations, or those of the publisher, the editors and the reviewers. Any product that may be evaluated in this article, or claim that may be made by its manufacturer, is not guaranteed or endorsed by the publisher.
